# Medical Miscellanies

**Published:** 1856-12

**Authors:** 


### *Medical Miscellanies.*

William Buckland, D. D., the celebrated geologist and Dean of Westminster, died on the 7th August, at Clapham, Eng. He was appointed to the Deanery of Westminster by Sir Robert Peel, in 1845. It is stated that shortly after his appointment, he became subjected to "the last infirmity of noble minds"—his intellect gave way. In November, 1846, it was our privilege to listen to his preaching, and sure we are that at that period he manifested no symptoms of the disease to which he afterwards became a victim.——The Library of the Royal College of Surgeons, England, contains over 30,000 volumes.——A new edition (the 7th) of Druitt's Surgery has recently been published in London.——According to the Boston Medical and Surgical Journal, Sept. 11th, the health, so long and seriously impaired, of Dr. J. Mason Warren, will soon be re-established. At present he meets his patients at his usual consultation hours, and has partially resumed his duties at the Massachusetts General Hospital. The Warrens are names inseparably associated with American Surgery.

Dr. Jas. Dubois, of New Utrecht, L. I., has fallen a victim to the yellow fever, which is prevailing in that vicinity. At the commencement of the epidemic, Dr. D. was advised by his friends to leave for a more healthy location, but he replied that it was his duty to remain, and not to desert the inhabitants in their hour of affliction. Like a true soldier, he died at the post of duty.

A quack in England has recently been convicted of manslaughter in causing the death of a patient by administering lobelia-inflata in five-grain doses. According to a statement made by Dr. Letheby, in 1853, Professor of Chemistry in the London Hospital, no less than thirteen cases of poisoning had occurred from this drug within the three or four preceding years, and in six of these a coroner's jury brought in a verdict of manslaughter. In the recent trial, the prisoner was sentenced to three months' imprisonment.

Dr. J. V. C. Smith, for many years editor of the Boston Medical and Surgical Journal, is about to establish a new journal, to advocate "more *liberal* principles."

In Mr. Marson's petition to the House of Commons on the Vaccination Bill, 1856, he mentions as an example of what can be done by efficient vaccination, the fact, that not one of the nurses or servants of the London Small-Pox Hospital has had small-pox for the last twenty years. They had all been either vaccinated or re-vaccinated at the commencement of their service.

Dr. H. D. Bulkley, formerly editor of the N. Y. Medical Times, has become associated with Drs. Purple and Smith, of the N. Y. Journal of Medicine, which is one of our best medical periodicals, and but too little known at the West. By this union of the editorial corps, both journals become consolidated. We can cordially recommend the N. Y. Journal to our readers.—*Western Lancet.*

### *Poisoning from swallowing Chloroform.*

The *Philadelphia Medical Examiner* contains an interesting case of death following the ingestion of about one ounce and a half of chloroform, diluted with about the same quantity of water. The patient was an intemperate woman, who swallowed the liquid by mistake, supposing it to be sweet spirit of nitre. The first symptoms were those of intoxication followed by insensibility, stertorous breathing, slow and feeble pulse, and great contraction of the pupils. She lived for about thirty-six hours, and died asphyxiated, having recovered her senses for several hours before death. The stomach was paler than usual, except in streaks a quarter of an inch in width, from which it was inferred that the organ had been thrown into folds by the irritation of the chloroform, so that only a portion of its surface had been acted on. The mucous membrane was much softened.—*Boston Med. and Surg. Journal.*

